# Evaluation of therapeutic effects of nanofibrous mat containing mycophenolate mofetil on oral lichen planus: In vitro and clinical trial study

**DOI:** 10.1080/26415275.2023.2283177

**Published:** 2023-12-23

**Authors:** Mahdieh Alipour, Ehsan Habibivand, Shayesteh Sekhavati, Zahra Aghazadeh, Mohammadreza Ranjkesh, Soghra Ramezani, Marziyeh Aghazadeh, Marjan Ghorbani

**Affiliations:** a Dental and Periodontal Research Center, Faculty of Dentistry, Tabriz University of Medical Sciences, Tabriz, Iran; b Stem Cell Research Center, Tabriz University of Medical Sciences, Tabriz, Iran; c Department of Oral Medicine, Faculty of Dentistry, Tabriz University of Medical Sciences, Tabriz, Iran; d Department of Dermatology, School of Medicine, Sina Medical Research & Training Hospital, Tabriz University of Medical Sciences, Tabriz, Iran; e Nanofiber Research Center, Asian Nanostructures Technology Co. (ANSTCO), Zanjan, Iran; f Department of Bioscience Research, Department of Medicine – Cardiology, Department of Microbiology, Immunology & Biochemistry, University of Tennessee, Tennessee, USA; g Nutrition Research Center, Tabriz University of Medical Sciences, Tabriz, Iran

**Keywords:** Aloe-vera, Nanofibrous mat, Oral Lichen Planus, Mycophenolate, Zinc oxide nanoparticles

## Abstract

**Objectives:**

Recently, topical drug delivery system has gained increasing interest in the treatment of oral lesions. Lichen planus is a chronic inflammatory disease affecting mucous membranes and skin. The current study aimed to fabricate a drug delivery system containing mycophenolate mofetil for the treatment of oral lichen planus lesions.

**Methods:**

Firstly, a nanofibrous mat containing mycophenolate mofetil, zinc oxide nanoparticles, and aloe vera was designed and fabricated. The antimicrobial, cytocompatibility, anti-inflammatory, and antioxidative characteristics of fabricated scaffolds were evaluated. Then, this nanofibrous mat was applied to 12 patients suffering from bilateral erythematous/erosive Oral Lichen planus (OLP) lesions for 2 weeks. The treatment outcomes, including oral symptoms and lesion size, were compared with the routine topical treatment of these lesions; Triamcinolone ointment.

**Results:**

The characterization of nanofibrous mat approved the successful fabrication of scaffolds. The fabricated nanofibers showed notable antimicrobial activity. The amounts of TNF 𝛼, IL6, and reactive oxygen species (ROS) of stimulated human gingival fibroblasts were decreased after exposure to NFs/Myco/Alv/ZnO scaffolds. The clinical trial results demonstrated the same therapeutic effects compared to the commercial ointment, while the symptoms of patients were significantly improved in the mats group.

*Significance.* Considering the successful results of this study, the application of nanofibrous mat can be a promising product for improving treatment outcomes of OLP.

## Introduction

Lichen planus is a chronic inflammatory disease affecting mucous membranes and skin. It has a worldwide prevalence rate of 0.2 percent to 2 percent (Patel et al. 2016). The etiology of this disease is unclear, but it is strongly related to damage of epithelial cells due to cytotoxic activity of CD8+ lymphocytes. The clinical manifestation of this mucocutaneous disease is sometimes limited to the oral mucosa, called oral lichen planus (OLP). Oral lesions are usually characterized in white papular and reticular forms; however other clinical manifestations includes plaque-like, erosive, atrophic, and bullous lesions (Lavanya et al. 2011). The presence of severe and multiple ulcers leads to challenging food consumption and daily life activities, followed by an extreme reduction of life quality (Daume et al. 2020). Although there is no definite treatment for OLP, the administration of topical and systemic corticosteroids is mainly considered. However, there is a lack of the desired effect because of the significantly short maintenance period in the oral cavity due to lack of mucosal adhesion to and saliva wash out (Lavanya et al. 2011; Said et al. 2021). To overcome these limitations, increased frequency of topical corticosteroids application is recommended (Gabros et al. 2020).

Currently, advanced topical therapies based on drug delivery systems with natural ingredients have attracted significant attention due to their considerable advantages. Compared to local applications or injection of corticosteroids, nanofibrous mate can adhere to oral mucosa and is more user-friendly (Shaikh et al. 2011). This type of treatment can be easily applied in unconscious and none cooperative patients while providing enough duration of drug durability and great concentration (Singh et al. 2017). Furthermore, in topical application, the side effects of systemic use such as nausea and vomiting are not observed; because an optimal concentration is topically used with a lower dose than systemic application (Samiee et al. 2020).

Different natural and synthetic polymers are applied to synthesize nano mates. Wheat contains gluten protein called gliadin, which exhibits bio-adhesive properties and has been tested as a topical or oral drug delivery system due to its biodegradable and biocompatible properties (Hajjari et al. 2021; Liu et al. 2018). Ethylcellulose is another natural hydrophobic and adherent biopolymer approved by the US Food and Drug Administration (FDA) for oral, vaginal, and ocular applications (Cazorla-Luna et al. 2020; Wasilewska and Winnicka 2019). Mycophenolate mofetil (Myco) is an immunosuppressive medication that has been successfully used to treat graft-versus-host disease (Frieling et al. 2003), lichen planus and rheumatoid disease (Iaccarino et al. 2007; Mutasim 2004; Samiee et al. 2020). Zinc (Zn) is an influential compound in the reduction of OLP symptoms such as pain, burning, and itching sensations (Chaitanya et al. 2019; Deveneni et al. 2020; Mehdipour et al. 2010). Aloe Vera is well known for its considerable medicinal benefits (Surjushe et al. 2008) and its Systemic and topical forms have been successfully used as an alternative treatment in OLP patients (Choonhakarn et al. 2008; Salazar‐Sánchez et al. 2010).

The present study aimed to develop a fibrous mats based on natural and FDA approved polymers containing available medications in the market as an alternative for topical treatment of OLP lesions. For this purpose Gliadin/Ethylcellulose-based mats were loaded with Myco, ZnO nanoparticles, and Aloe Vera. The fibers were characterized in terms of chemical and mechanical properties, and their biocompatibility, anti-inflammatory, anti-oxidant effects were evaluated in the *in-vitro* phase. Finally, its clinical effects were assessed as a blind randomized clinical trial on patients with bilateral oral lichen planus lesions and compared with the routine topical treatment.

## Materials and Methods

EC, Gliadin (average MW: 150 kDa), and Muller Hinton Agar were obtained from Sigma-Aldrich, USA. Ethanol (97% v/v purity) was purchased from Merck Co., Germany. ZnO powder was obtained from Iranian Nanomaterials Pioneers Co. (range of particle size 10–30 nm). Escherichia coli (PTCC 1163) and Staphylococcus aureus (PTCC 1764) were purchased from Persian Type Culture Collection (PTCC). Alv powder was acquired from Melbourne, Florida, USA Company. Myco was prepared from EMC Co-UK (ZTC code: L04AA06). The protocol of this study complied with the Helsinki declaration and was approved by Tabriz University of medical science with the ethical code of IR.TBZMED.REC.1399.339. Also, the study protocol was registered in the Iranian Registry of Clinical Trials Center with the number IRCTID: IRCT20200708048057N1, and with URL: www.IRCT.IR. All of the materials used in the study were either FDA approved or over the counter medication.

### Preparation of electrospun nanofibers

Ethylcellulose/gliadin control solution (ratio 60/40) was prepared in ethanol 70% v/v solvent with a concentration of 22 w/v%. Moreover, the polymer solution containing active ingredients of Alv, ZnO NPs, Myco with a concentration of 10, 2, 2% wt (versus total polymer weight) was prepared. The prepared solutions were loaded into a 5 mL plastic syringes equipped with a blunt-tipped 21-gauge stainless steel needle. Then, the electrospinning was carried out by applying a high voltage power of 18 kV and a flow rate of 1.0 ml/h. Nanofibers were collected on aluminum foil on a rotating drum with a speed of 500 rpm at a distance of 15cm from the needle tip.

### Characterization tests

The morphology and size distribution of nanofibers were considered using scanning electron microscopy (SEM, LEO960, Denmark). The samples were gold sputter-coated for 60 s under argon prior to visualization. The diameters of samples were analyzed by ImageJ software (Media Cybernetics Ltd., USA). The nanofibers were dried before the experiment, and the Fourier Transform Infrared Spectrometer (FT-IR, Bruker-Tensor27) was used to determine the functional groups of the developed nanofibers. The spectra were recorded at a resolution of 4 cm^−1^ in the range of 4000-500 cm^−1^. The nanofibrous mat’s water contact angle (WCA) was measured using a 2 μL of deionized water drop on the surface of nanofibers and (KSV Instruments Ltd.) contact angle instrument. The WCA of each droplet was measured using an automatic optical contact measuring meter. Thermo Gravimetric Analysis (TGA) was carried out (Germany, Linseis, STAPT1600) to analyze the thermal stability of nanofibers. The N_2_ atmosphere at a heating rate of 10 °C/min was applied. A general testing machine (Shimadzu 20KN-testing machine, ASTM D256) with 5 mm.min^- 1^ crosshead speed was used to evaluate the mechanical properties of nanofibers with 30 × 10 mm^2^ dimensions. To study the *in-vitro* degradation, the samples were weighed (W_1_), immersed in PBS and incubated at 37 °C for 3, 7, 10, 14, 20, and hours in three different PH. After these times, the samples were removed from the solution and weighed (W_2_). The weight loss (WL) was calculated by the following Eq:

$$\text{WL}\;\left(\text{\%}\right)=\frac{{\text{W}}_{1}\;-\;{\text{W}}_{2}}{{\text{W}}_{2}}\times 100$$

### Drug Release Profile

To measure drug released from nanomates, 50 mg of samples were dispersed in phosphate-buffered saline (three different PH= 5.3, 7.4, 8.6) at 37"°C and poured into test tubes with gentle agitation. One test tube was used for quantifying the release amount of mycophenolate mofetil at a defined time interval to avoid inaccuracy in the removal of solution and addition of buffer. After the incubation period, each sample was centrifuged, and the supernatant was withdrawn from the incubation medium and measured for mycophenolate mofetil concentration by UV spectroscopy at 250 nm.

### MTT assay and Cell adhesion

Human Gingival Fibroblasts (HGF) were purchased from the Pasteur Institute of National Cell Bank (C-165; NCBI, Tehran, Iran) and were cultured on sterilized blank NFs and Myco/NFs/Alv/ZnO respectively as control and experimental group. The scaffolds were placed in 96-well plates and put under UV light for 20 min; then, trypsinized HGF were seeded in numbers of 10,000 cells per well. To evaluate the viability of these cells, an MTT assay was performed after 1, 3, and 5 days of cell seeding as described previously (Alipour et al. 2019).

Cell adhesion, penetration, and proliferation were evaluated with scanning electron microscopy (SEM, LEO960, Denmark). For this purpose, 1, 3, and 5 days after seeding of HGFs on each scaffold, the samples were fixed by 2.5% glutaraldehyde solution followed by gradient drying by ethanol as described previously (Alipour et al. 2022; Alipour et al. 2021).

### Anti-inflammatory and antioxidative tests

For evaluating anti-inflammatory and antioxidative properties of the synthesized nanofibrous mat, the seeded cells were inflamed with 10 ng/ml LPS of *E. Coli* (Alipour et al. 2021). Cells with and without LPS were considered as negative and positive control groups. According to the manufacturer’s protocol, the concentration of TNF *𝛼* and IL6 was measured using ELISA Kits (Duoset ELISA Development Kit, Cat No. Dy 210-05). For measuring the cellular reactive oxygen species (ROS) levels in each experiment group, the DCFDA-ROS detection assay kit (Sigma-Aldrich Co., USA; Cat No. MAK142) was used (Alipour et al. 2021).

### Antimicrobial tests

For assessment of antimicrobial properties of the fabricated nanofibrous mat, the agar diffusion test was performed against *S. aureus* and *E. Coli*. Briefly, 1.5 × 10^8^ CFU.ml^−1^ of bacterial suspension were prepared and cultured on the surface of the Mueller Hinton Agar Plates. The fabricated nanofibrous mats were cut in a circular shape of 5mm diameter. The samples were placed on the surface of plates and incubated at 37 °C. After 24 hours, the dimension of the inhibition zones around the blank Nfs and Myco/Nfs/ZnO/Alv was measured with a caliper.

### Clinical assessment

**Figure 1.**
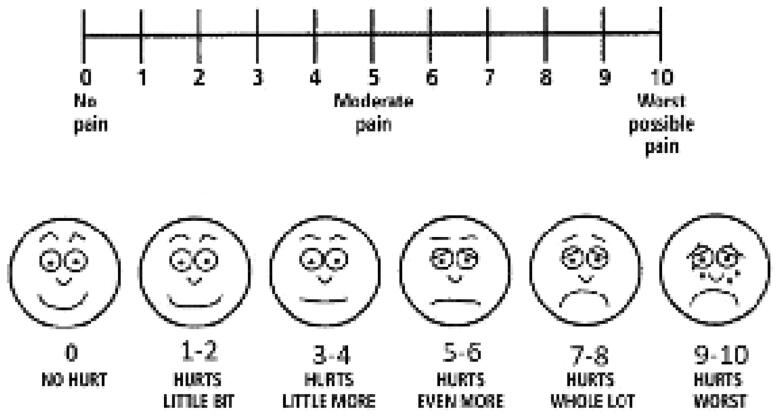
VAS scale; used for assessment of the severity of pain and burning sensations by patients

**Figure 2.**
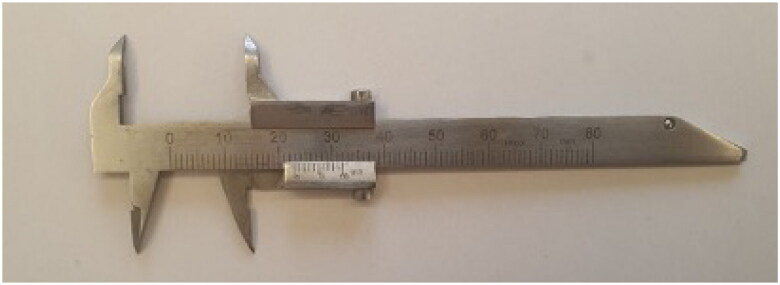
Digital calibrated caliper; used for size assessment of oral lesions before and after treatment

This clinical trial was performed on 12 patients suffering from bilateral erythematous/erosive OLP lesions and referred to the Oral Medicine Department of Tabriz University of Medical Sciences. The subjects were selected from the Patients over 18 years old whom their bilateral erosive OLP disease was confirmed by clinical and pathological examination and willing to participate in the study signing the consent form. Patients receiving drugs affecting the immune system in the past one month and patients with contraindications of administration of topical corticosteroids such as uncontrolled diabetes, tuberculosis, and gastric ulcer were excluded from the study (Mehdipour et al. 2010). Firstly, all participants underwent a general health check by a dermatologist using a questionnaire to ensure that the symptoms were limited to the mouth and no dermal lesions existed. Then, oral lesions of each side were examined, and the checklists for the lesions size and symptoms were filled. To evaluate the healing process of OLP lesions, two factors were considered in the checklists:

1. Severity of pain and burning sensation assessment by visual analog scale (VAS) (Figure 1).
2. Changes in lesion size evaluated by digital calibrated calipers (Figure 2).

**Figure 3.**
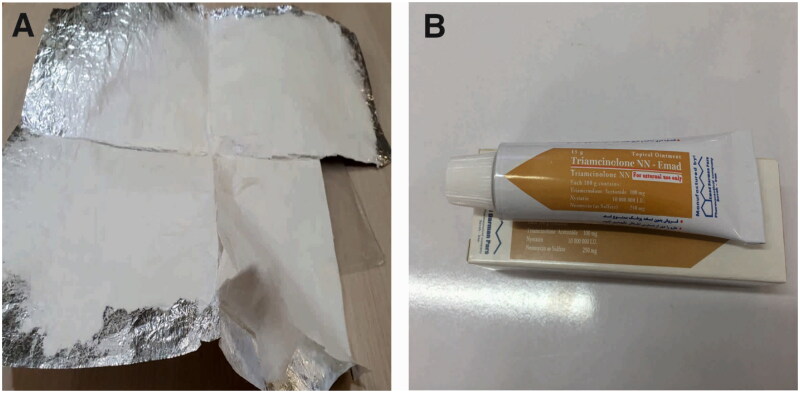
**A)** Synthesized Myco/NFs/ZnO/Alv nanofibrous mat, **B)** Commercial Triamcinolone Ointment

VAS scale is a 10 cm long line that is graded from zero to 10 measuring patients’ pain and burning sensation severity. Number zero indicated the absence of pain and burning sensation, and number 10 indicated the maximum amount of them. At the first visit, each patient marked a distinct point on this line based on his pain and burning sensation intensity in each side. Wound size was measured by a digital caliper (Guanglu) at two dimensions (maximum length and width). These processes were repeated in the second visit; two weeks later. All of the patients received two types of medications; each was considered to be applied on one side of oral lesions, nanofibrous mats containing Myco, ZnO NPs, and Aloe Vera (Figure 3, A), and the routine topical treatment for OLP lesions; Triamcinolone acetonide 0.1% ointment (Triamcicort N.N. Topical ointment, Emad Darman Pars, pharmaceutical Co, Saveh, Iran) (Figure 15. B). To make the study blind, the examiner was unaware of the applied treatment on each side.

Nanofibrous mats were provided in 2 × 2 or 1 × 2 cm pieces. These mats were sterilized by gamma radiation and were storable at room temperature. Patients were recommended to use the medications after breathing with the mouth for 30 seconds until the lesion’s surface was dried. The patient was instructed to abstain from eating, drinking, and rinsing for an hour. This process was applied twice a day in the morning after breakfast and in the evening after dinner.

### Statistical analysis

All collected data were statistically analyzed by Prism software (version 8.0, GraphPad, San Diego, CA, USA) for cell studies. Clinical data were analyzed using SPSS 26 (IBM). The Mann-Whitney U test and paired t-test were used for statistical analysis of the clinical section. Statistical significance was set at P < 0.05.

## Results

### Characterization of developed nanofibers

#### SEM

**Figure 4.**
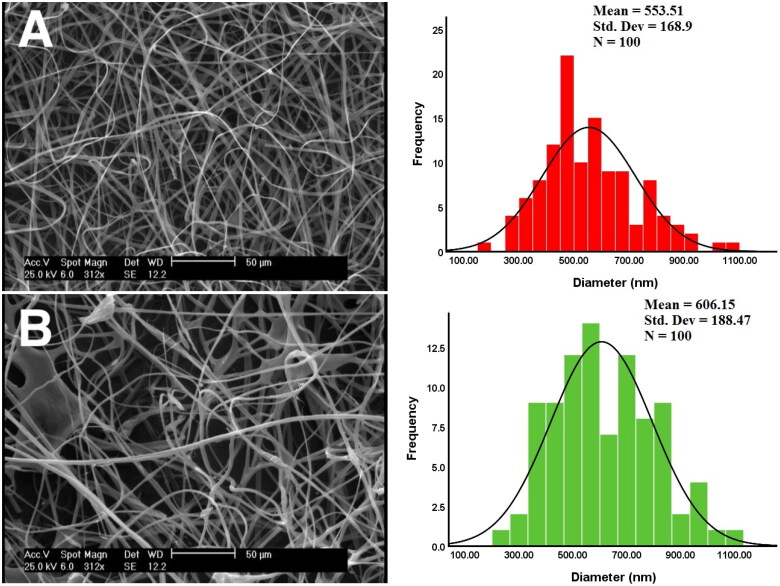
Scanning electron microscopy (SEM) images of fabricated nanofibers and average diameter. A) Blank nanofibrous mat, B) Myco/NFs/Alv/ZnO nanofibrous mat.

According to the SEM images (Figure. 4), the blank nanofibers exhibit uniform fibers, with no visible beads and ribbon-like fibers. A mean diameter of 553.5 ± 168nm was observed for blank nanofibers. Myco/NFs/Alv/ZnO shows gradual morphological changes with some beads however, regular cylindrical fibers were observed. This formulation controlled a range of fiber diameters with the mean diameter lying at 606.15 ± 188 nm.

#### FTIR

**Figure 5.**
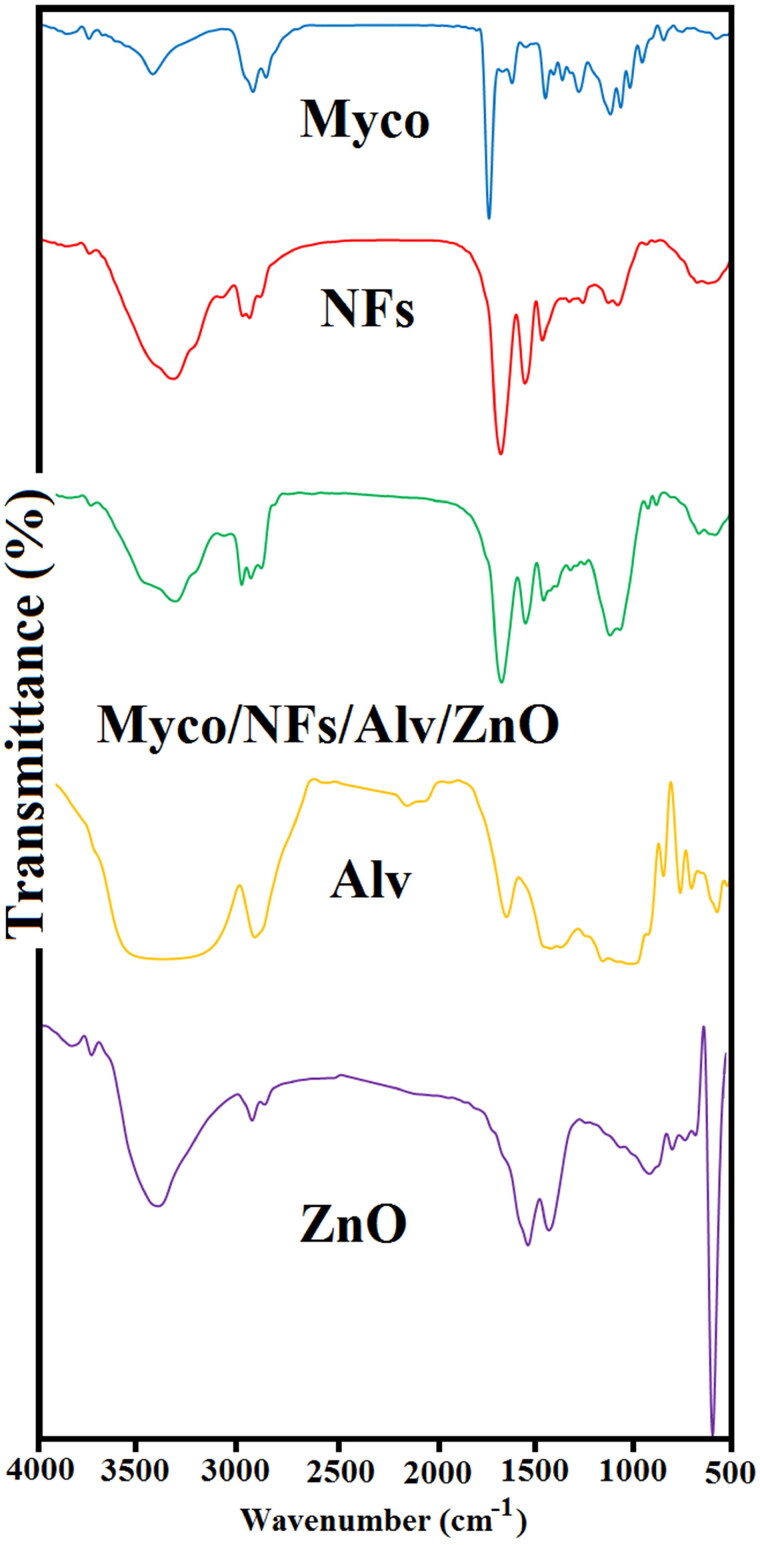
Fourier transform infrared spectroscopy (FT–IR) spectra of Mycophenolate mofetil (Myco), Blank NFs, Myco/NFs/Alv/ZnO Gliadin/Ethylcellulose, Alv, and ZnO.

Figure. 5 presents the FT-IR spectrum of samples. EC/Gliadin nanofibers showed the characteristic peaks at 1052 and 1315 cm^−1^, indicating C-O-C stretching and -CH bending, respectively(Khichar et al. 2020). Bands at 2972 and 2870 cm^−1^ arise from -CH stretching vibrations. These peaks are observed in Myco/NFs/Alv/ZnO formulations without any significant changes in peak positions. In the spectrum of Alv, the absorption peak of about 3311 cm^−1^ was related to the hydrogen bonds of N-H groups (Mohebian et al. 2022). Moreover, the absorption peak at 2910 cm^−1^ corresponded to the symmetrical and asymmetrical C-H stretching of the -CH_2_ groups (Ghorbani et al. 2020). The absorption peak confirmed the presence of carbonyl groups at 1710 cm^−1^ (Mohebian et al. 2022).The stretching vibrations of C = O groups of esters and phenols were observed at 1240 cm^−1^ (Alvandi et al. 2022). The peak at 581 cm^−1^ was the characteristic absorption of the Zn–O bond and other peaks attributed to the carboxylate and hydroxyl impurities in the materials (Hedayati 2015). The IR spectrum of pure Myco was also recorded (Iqbal et al. 2020). The asymmetric stretch of N-O at ranges from 1475–1550 cm^−1^ proved the presence of nitro compounds. The stretching region of carbonyl groups ranged from 1710–1770 cm^−1^ representing carboxylic acids (Pătruţescu et al. 2015). C-H band of alkenes and C-N stretch of aliphatic amines were observed between the wavelengths of 800-1000 cm^−1^ and 1020–1250 cm^−1^, respectively (Pătruţescu et al. 2015).

### Mechanical properties

**Figure 6.**
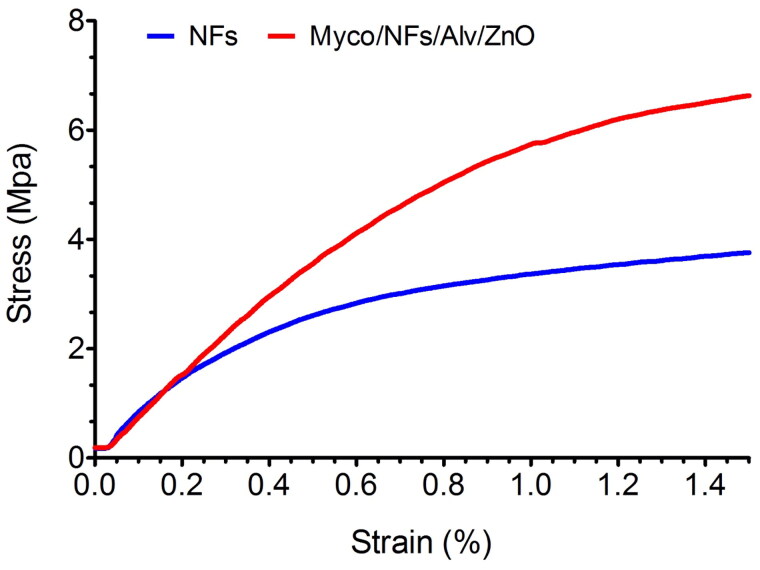
Tensile strength of NFs and Myco/NFs/Alv/ZnO.

The mechanical properties of two nanofibers were further assessed, and the representative tensile stress-strain curves were exposed in Figure 6. The tensile strength of blank nanofibers and Myco/NFs/Alv/ZnO were 3.75 ± 0.2 MPa, and 6.63 ± 0.33 MPa, respectively. The tensile strength was remarkably enhanced after incorporating Myco/Alv/ZnO. The results demonstrated that the mechanical property of Myco/NFs/Alv/ZnO was significantly (P < 0.05) higher than those in blank nanofibers due to the formation of hydrogen bonds between the EC/Gliadin and ZnO NPs, Alv and Myco (Table 1.) that led to the increasing the overall crystallinity and the compactness of polymer chains (Amjadi et al. 2020).

**Table 1.** Tensile strength (MPa) of Blank NFs and Myco/NFs/Alv/ZnO.

Samples	Tensile strength
Blank NFs	3.75 ± 0.2 MPa
Myco/NFs/Alv/ZnO	6.63 ± 0.33** MPa

Values are presented as mean ± SD. **P shows significant differences (P < 0.05) compared to Blank NFs.

#### WCA

**Figure 7.**
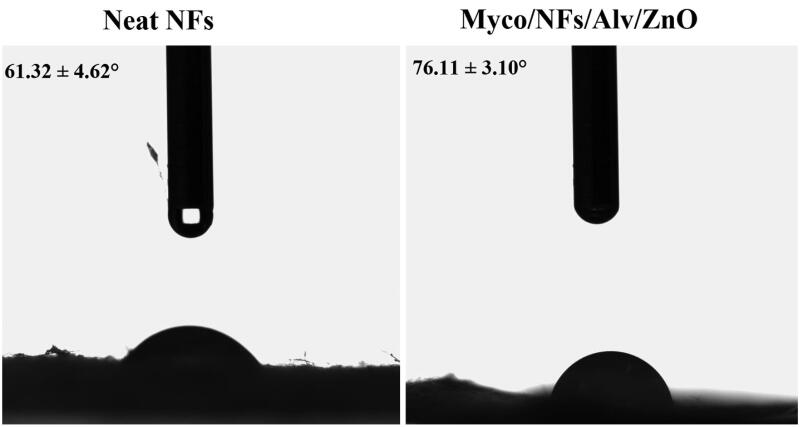
Water contact angles Electrospun Blank NFs and Myco/NFs/Alv/ZnO scaffolds.

The water contact angles of the blank nanofibers and Myco/NFs/Alv/ZnO were analyzed (Figure. 7). The results demonstrated that the water contact angle of the blank nanofibers increased from 61.32° ± 4.62 to 76.11°±3.10 after the incorporation of Alv, ZnO NPs, and Myco due to the hydrophobic nature of these two compounds.

#### TGA

**Figure 8.**
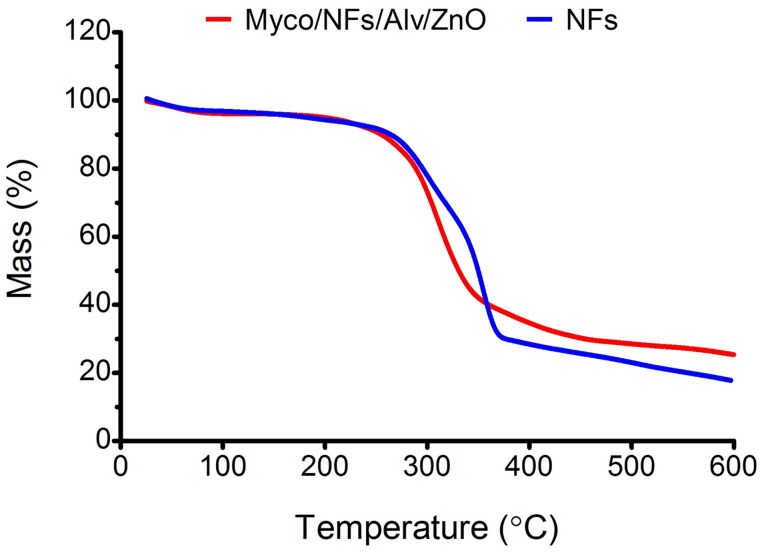
TGA curves of blank NFs and Myco/NFs/Alv/ZnO nanofibrous mat.

The TGA curves of the samples were shown in Figure. 8. In this regard, the first stage of weight loss in the range of 50–250 °C was ascribed to the vaporization of bound water and the loss of acetyl moieties from the backbone of EC, while the second stage over a temperature range of 250–600 °C was related to the EC/Gliadin degradation and the break of intermolecular bonds (Wang et al. 2020). It should be noted that the figures showed the better thermal stability of Myco/NFs/Alv/ZnO than blank nanofibers owing to the stronger molecular interactions between Myco, Alv, ZnO, and nanofibers facilitated the formation of a denser network, resulting in higher residues left for Myco/NFs/Alv/ZnO. Therefore, the residual weights for Myco/NFs/Alv/ZnO at a temperature of 600 °C were higher than those of blank nanofibers. This result could be attributed to the formed interaction through the hydrogen bonds (Luo et al. 2021).

### *In Vitro* Degradation

**Figure 9.**
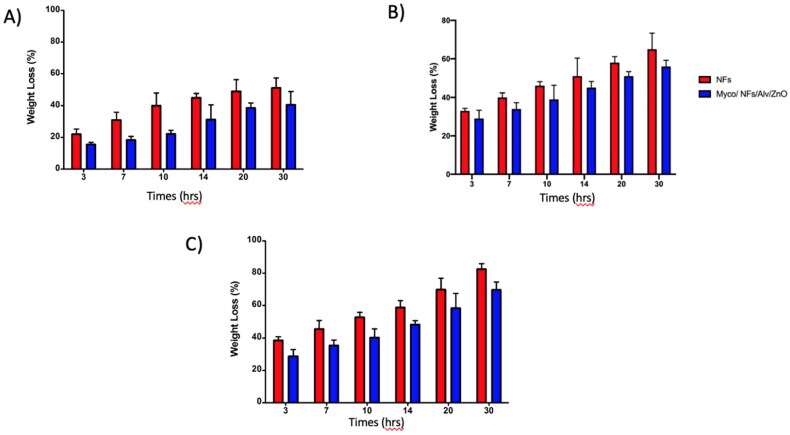
Weight loss of blank NFs and Myco/NFs/Alv/ZnO nanofibrous mat in different PH; A) PH = 4, B) PH = 7, C) PH = 9.

According to the results (Figure 9) the degradation rate of blank nanofibers in all PHs was higher than that of Myco/NFs/Alv/ZnO. In Nutral PH, after 30 hours, the residual weight of blank nanofibers was ∼22%, while this value was ∼35% for Myco/NFs/Alv/ZnO. Therefore, a slow degradation rate was observed for Myco/NFs/Alv/ZnO, which had a higher fiber diameter.

### Drug Release Profile

**Figure 10.**
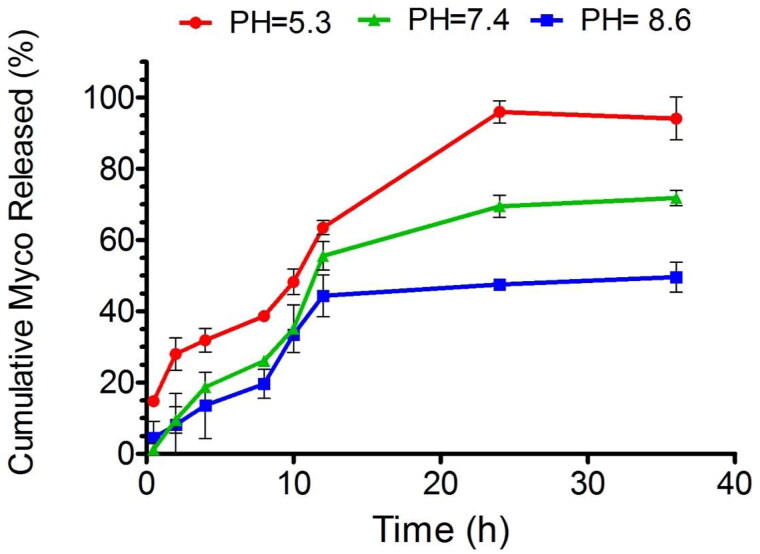
The release pattern showed that less than 20% of muco is released in different PH during first hour.

Given the differing pH environments within the oral cavity under various conditions, encompassing acidic, basic, and neutral states, our study focused on assessing the release dynamics of myco within these contexts. Notably, our findings revealed that across all three conditions, the initial hour saw a release of myco of less than 20% (Figure 10).

### Cell viability and cell adhesion

**Figure 11.**
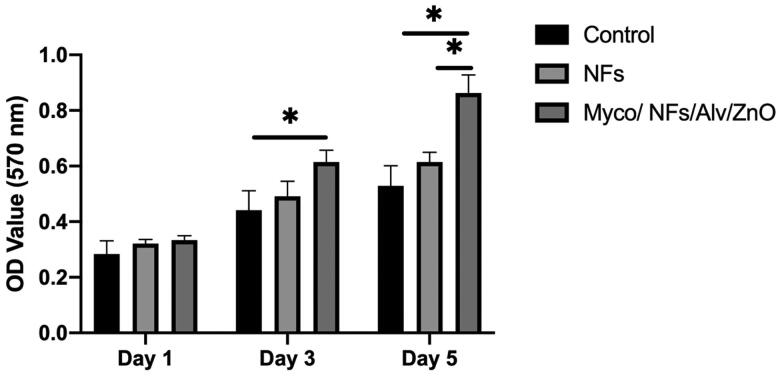
MTT assay results for human gingival fibroblast cells (HGF) cells, NF, and Myco/NFs/Alv/ZnO nanofibrous mat.

The cytocompatibility of the fabricated scaffolds and adhesion of Human Gingival Fibroblasts (HGF) on the fabricated nanofibrous mat showed that the proliferation of HGF on Myco/NFs/ZnO/Alv was higher during all experimental days; this difference was significant on days 3 and 5 (Figure 11).

**Figure 12.**
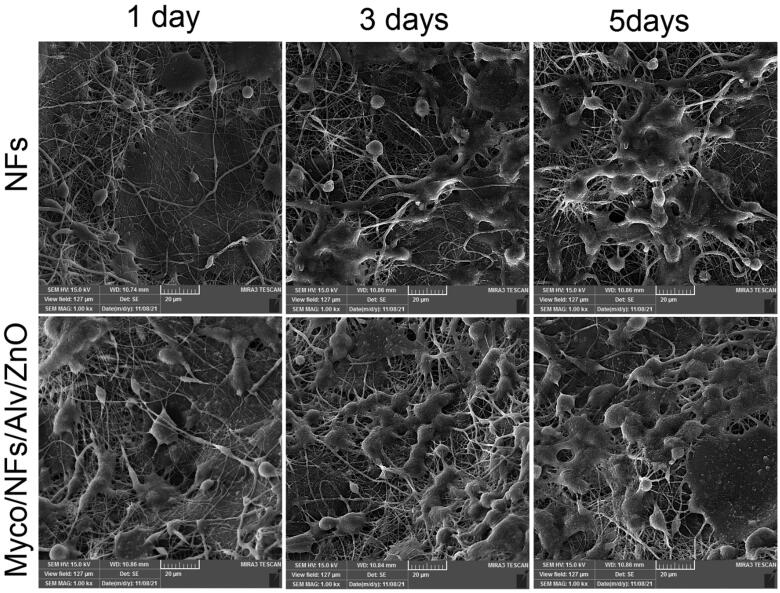
The SEM images of human gingival fibroblast cells (HGF) adhered on the surfaces of **A)** NFs, and **B)** Myco/Nfs/ZnO/Alv nanofibrous mat after 1, 3, and 5 days.

In adhesion, penetration, and proliferation assay of HGFs by SEM it was revealed that cells were attached and proliferated in Myco/Nfs/ZnO/Alv more than blank Nfs (Figure 12). This finding suggests better interaction between cells and fabricated nanofibers. The MTT and SEM evaluation results indicated that Myco/Nfs/ZnO/Alv provides appropriate structure for cell adhesion, penetration, and proliferation without any cytotoxic effects.

### Anti-inflammatory and antioxidative studies

**Figure 13.**
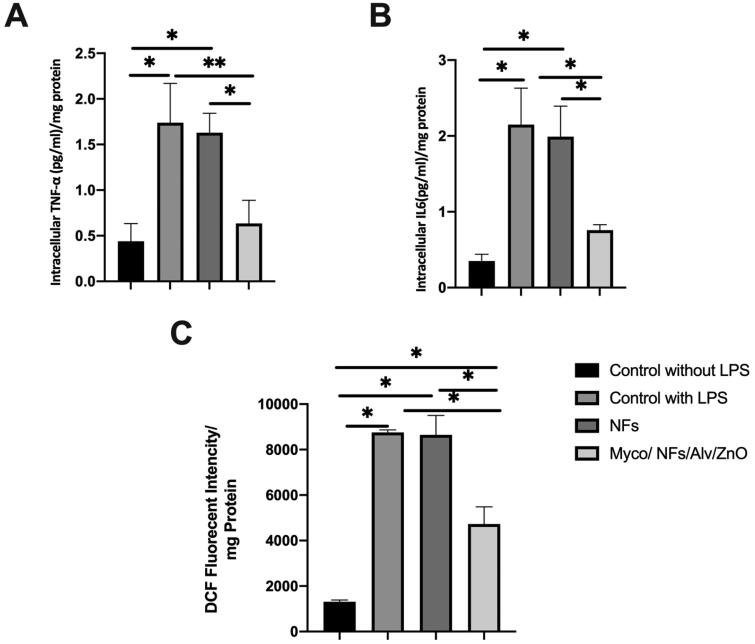
**A)** Intracellular TNF *𝛼,* **B)** Intracellular IL6, and **C)** DCF levels in the experimental groups.

The anti-inflammatory effects of the synthesized Myco/Nfs/ZnO/Alv nanofibrous mat caused to significantly reduction in the level of IL6 and TNFα (Figure 13).

**Figure 14.**
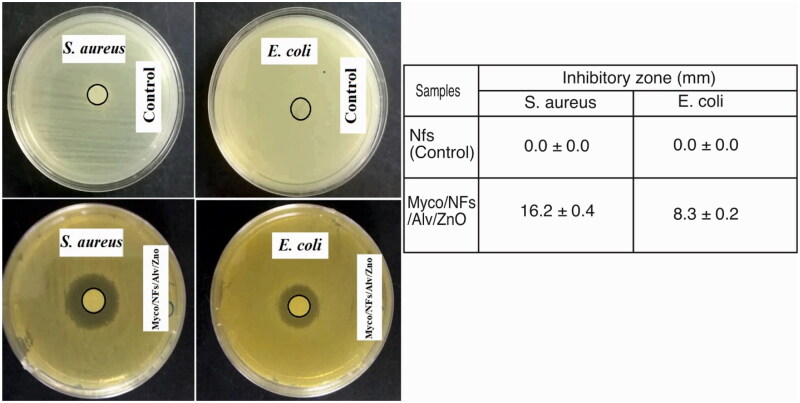
The antibacterial activity of electrospun NFs against *S. aureus* and *E. coli* bacteria. Data are expressed as mean ± standard deviation (n = 3).

For antioxidative properties, dichlorofluorescein (DCF) levels were measured (Figure 13.C). The fluorescent intensities of this marker were significantly decreased in the experiment group.1307.86, 8754.40, 8648.37, and 4727.12 in the negative control group, positive control group, blank NFs, and Myco/Nfs/ZnO/Alv group, respectively.

### Antimicrobial test

The antimicrobial properties of fabricated nanofibers against both Gram-positive (*S. aureus*) and Gram-Negative (*E. Coli*) bacteria revealed that Myco/Nfs/ZnO/Alv nanofibers showed significantly antimicrobial activity against selected bacteria (Figure14).

### Clinical trial study

**Figure 15.**
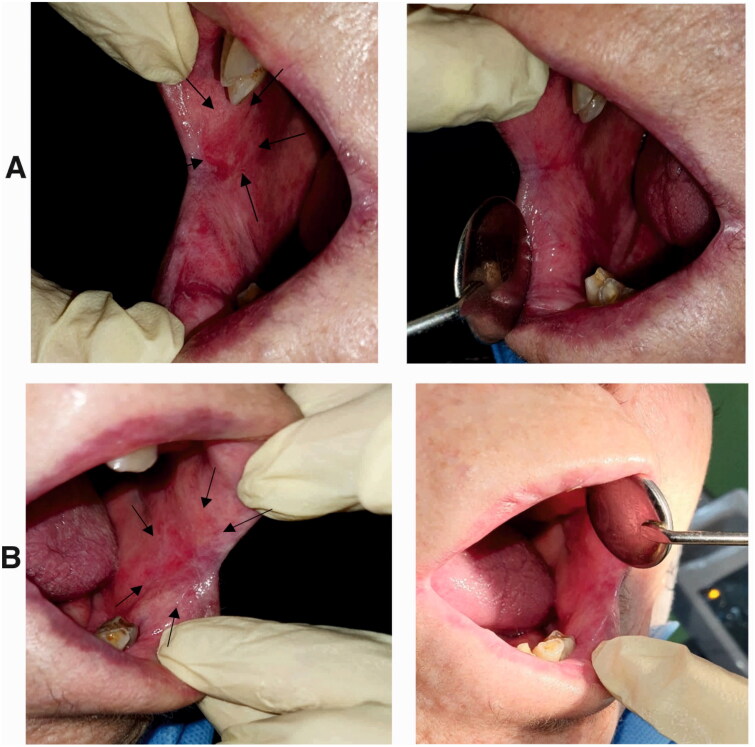
**A)** Erosive OLP lesions in a patient (right buccal mucosa) treated with Triamcinolone ointment. Left: before treatment, an oval shape erosion was obvious in the buccal mucosa next to the commissure of the lips. Right: after treatment. The erosive lesion was completely removed, and the healthy mucosa was regenerated. **B)** Erosive and reticular OLP lesions in the same patient (left buccal mucosa) treated with nanofibrous mats. Left: before treatment, an oval shape erosion with white lace-like reticular lines was obvious in the buccal mucosa next to the commissure of the lips. Right: after treatment. The erosive lesion and most of the white reticular lines were removed.

**Figure 16.**
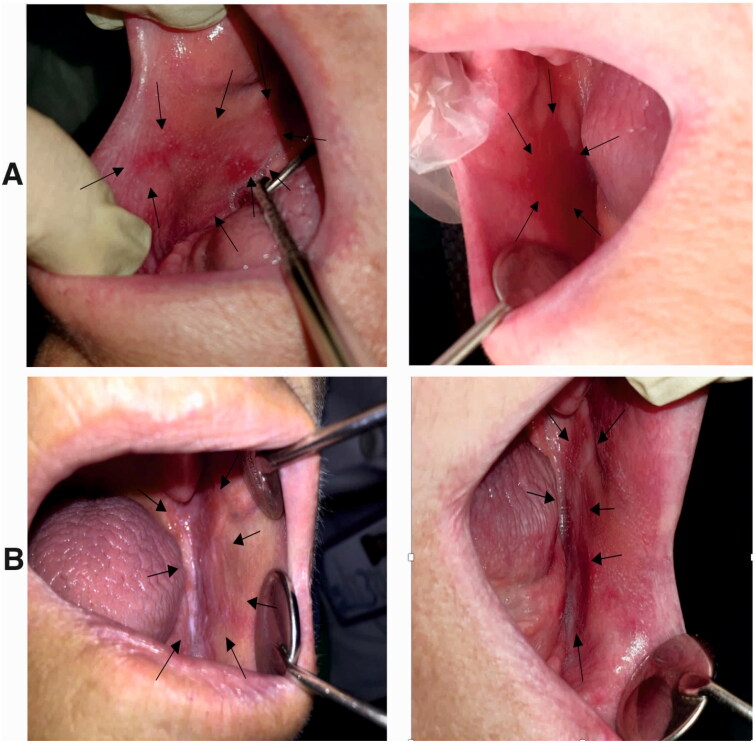
**A)** Erosive lesion extended in the buccal mucosa of an edentulous patient (right buccal mucosa). This lesion was treated with triamcinolone ointment for two weeks, and the size of the lesion was decreased after treatment. Left: before treatment, the lesion was almost extended throughout the length of buccal mucosa from the commissure of the lips to the ramus. Right: after treatment, the size of the lesion was decreased after receiving the treatment. **B)** Erosive and reticular lesions of OLP in the left side of the same patient. Right: before treatment, the lesion was extended in the buccal mucosa and the mandibular ridge. Left: after treatment, these lesions were received nanofibrous mats, and after two weeks, the reticular form was almost disappeared, and slight erosion was observed in the distal of buccal mucosa next to the ramus.

In this study 12 patients were included. One patient left the study due to long-distance which made traveling for follow-ups difficult, and the second was excluded because of confusing the sides of treatment during the treatment period. Ten patients (6 female and 4 male) at the mean age of 47.8 years successfully completed the two weeks treatment (Figure 15 and 16).

At the baseline, the mean size of the erosion in the nanofibrous mat group (case group) was 29.8×15.7 mm, while the mean size of lesions in the triamcinolone group (control group) was 33.4×15 mm. There was no significant difference between the sizes of the bilateral lesions in patients before the treatment.

After two weeks, the mean size of the lesions was decreased to 15.5×5.7 in the case and 19.1×7.9 mm in the control group. Comparing the two groups with each other, there was no significant difference in reducing the size of the lesions. This data clarified that the nanofibrous mats were as effective as the routine treatment for OLP lesions (Table 2).

**Table 2.** The mean size of the lesions both in length and width before and after treatment in nanofibrous mats and triamcinolone ointment groups. According to these data, the mean size of the lesions was decreased in both groups. The difference was not significant between the two studies groups. Hence, the nanofibrous mats were as effective as triamcinolone ointments.

Studied groups	Number of patients	Nanofibrous mats (Case)	Triamcinolone ointment (Control)	P-value
Mean size of lesions before treatment, Mean ± SD (mm)	10	Length: 29.800 ± 3.942	Length: 33.400 ± 5.9175	0.1268
		Width: 15.700 ± 2.3101	Width: 15.000 ± 3.5245	0.6058
Mean size of lesions after treatment, Mean ± SD (mm)	10	Length:15.500 ± 3.7781	Length: 19.100 ± 3.9781	0.0526
		Width: 5.70 ± 2.9824	Width: 7.900 ± 3.6460	0.157

Before treatment, the median VAS score in the nanofibrous mat group was 6.1, while in the triamcinolone group it was 5.6. Comparing these intensities by independent t-test showed that there was no significant difference between the intensity of pain and burning sensation between the two groups in the beginning of study (P= 0.7763). After completing the treatment period, the VAS was decreased to 2.7 and 3.7 respectively in the case and control groups. The statistical analysis showed a significant difference between two groups in the end of study (P = 0.0416). These data demonstrated that based on the patients’ viewpoints, the application of nanofibrous mats was more effective than triamcinolone ointments in reducing OLP symptoms (Table 3). There were no reports about the adverse effect of the medications by patients.

**Table 3.** Comparison of mean VAS scores between two studied groups before and after treatment. This data demonstrated that burning and pain sensation intensity was reduced in both groups. The VAS score after treatment in nanofibrous mat group was significantly less than the control group, confirming that this treatment was more effective in removing OLP symptoms from patients’ viewpoints.

Studied groups	Number of patients	Nanofibrous mats (case)	Triamcinolone ointment	P-value
Mean VAS scores before treatment	10	6.1 ± 1.663	5.6 ± 1.429	0.7763
Mean VAS scores after treatment	10	2.7 ± 0.949	3.7 ± 1.085	0.0416

## Discussion

As a prodrug of mycophenolic acid, mycophenolate mofetil (Myco) inhibits the two isoforms of inosine monophosphate dehydrogenase.Mycophenolic acid has a cytotoxic effect on lymphocytes. As a result, Myco is a valid treatment option for a number of autoimmune skin conditions (Didona et al. 2022)

Some studies have reported its effectiveness, but overall, there is weak evidence to support its routine use in OLP. With all this the topical consumption of myco is mentioned in some guidelines, which demonstrates the possible effective role of this medication in autoimmune mucocutaneous disease (Dalmau et al. 2007).

The present study developed a Gliadin/Ethylcellulose nanofibrous mat loaded with Myco, ZnO NPs, and Aloe Vera as anti-inflammatory, immunomodulatory, and reparative components. As best knowledge of the authors, this was the first time that the combination of Myco, ZnO NPs, and Aloe Vera was loaded on fibrous mats and successfully applied for the treatment of OLP lesions. After characterizing the chemical and mechanical properties of the fibers, biocompatibility, cell attachment, anti-inflammatory, anti-microbial, and anti-oxidant tests were performed. Then, its therapeutic effects on OLP lesions were evaluated in a blind clinical trial comparing with Triamcinolone acetonide ointment. The fabricated Gliadin/Ethylcellulose fibrous mats containing Myco, ZnO NPs, and Aloe Vera demonstrated smooth, uniform morphology and suitable mechanical properties for oral ulcer dressing. According to our results, these nanofibrous mats did not show any cytotoxic effects. Moreover, there was a significant reduction in TNFα, Il-6, and ROS levels in the Myco/NFs/ZnO/Alv group after stimulation of HGF with bacterial LPS. The microbial tests revealed that applying these nanofibers in the culture plates inhibited the growth of *S. aureus* and *E. coli*. Furthermore, in a two-week blind clinical trial, the fibers were administrated in patients with diagnosed bilateral erosive OLP lesions. After two weeks, the OLP signs and symptoms were significantly improved in the nanofibrous mat sides according to the VAS scale and lesion size measurement. The size of the lesions was decreased both in length and width, as well as the triamcinolone side. These results showed that nanofibers’ application was as effective as the routine treatment for these lesions.

In a similar randomized clinical trial, nanofibrous mats containing 2%Myco were used in twenty-seven patients with OLP lesions for 4 weeks (Samiee et al. 2020). The results showed that the mucoadhesive patch had positive effects on the healing of lesions and the burning sensation of the patients. Although this result was time-dependent and there was no significant difference up to three weeks, the patch application was reported successful compared to the placebo (Samiee et al. 2020). Our study used ZnO NPs and Aloe Vera as reparative and anti-inflammatory components besides Myco, an immunosuppressive medicament. Our results showed a shorter healing time for these lesions, and the clinical manifestation of the patients was improved in two weeks.

In a retrospective review of 10 patients with severe ulcerative oral lichen planus, systemic treatment with myco demonstrated the efficacy and favorable side-effect profile (Wee et al. 2012).

The presence of zinc plays a crucial role in epithelial growth and development (Uwitonze et al. 2020). It is an over the counter supplement and a critical element of cellular function and metabolism. Lower serum zinc level was reported in patients with erosive OLP lesions (Gholizadeh et al. 2014). Furthermore, several studies suggest the administration of Zinc mouthwashes or Zinc sulfate supplementation along with the main medications as a supportive treatment for oral ulcer healing (Mehdipour et al. 2016; Orbak et al. 2007; Oshvandi et al. 2021).

Aloe Vera was the other main component of the fabricated nanofibrous mats in this study. This material is used in toothpaste and oral mouthwashes due to its antimicrobial and anti-inflammatory properties (Ahmadi 2012; Karim et al. 2014; Korkmaz et al. 2019; Namiranian and Serino 2012). Recently, it was used in the form of probiotics for patients with recurrent aphthous stomatitis. Besides returning oral bacteria to normal levels in these patients, the gel reduced the healing time (Shi et al. 2020).

In the present study, the anti-inflammatory, antioxidant and antimicrobial effects of the nanofibrous mat containing Myco, ZnO NPs, and Aloe Vera were observed in the in vitro phases and confirmed by the clinical trial results. Considering the successful results of this study, the developed nanofibrous mat might have positive prospects in clinical applications. However, this study is a primary investigation with a limited sample size, emphasizing the necessity for further research in this area to yield comprehensive insights.

## Data Availability

All data generated and/or analyzed during this study are included in this published article. The datasets used and/or analyzed during the current study are available from the corresponding author on reasonable request.

## Abbreviations

OLP: Oral Lichen Planus
Myco: Mycophenolate
ZnO: Zinc oxide
Alv: Aloe-vera
NF: Nanofibrous mat
